# Performance of Tuberculin Skin Test Measured against Interferon Gamma Release Assay as Reference Standard in Children

**DOI:** 10.1155/2014/413459

**Published:** 2014-02-10

**Authors:** Michael Eisenhut, Katy Fidler

**Affiliations:** ^1^Paediatric Department, Luton & Dunstable University Hospital NHS Foundation Trust, Lewsey Road, Luton LU4 ODZ, UK; ^2^Department of Infection and Immunology, Brighton & Sussex Medical School, University of Sussex, Brighton, East Sussex BN1 9PX, UK

## Abstract

*Objectives*. International guidelines differ in the threshold of tuberculin skin test (TST) induration regarded as indicating *Mycobacterium (M.) tuberculosis* infection. Interferon gamma release assay (IGRA) results were used as reference to assess performance of TST induration thresholds for detection of *M. tuberculosis* infection in children. *Design*. Systematic review which included studies containing data on TST, IGRA, and Bacillus Calmette-Guérin (BCG) status in children. Data bases searched were PubMed, EMBASE, and the Cochrane library. Specificities and sensitivities were calculated for TST thresholds 5, 10, and 15 mm and correlated with age and geographical latitude. *Results*. Eleven studies with 2796 children were included. For BCG immunised children diameters of 5, 10, and 15 mm had median sensitivities of 87, 70, and 75% and specificities of 67, 93, and 90%, respectively. In non-BCG immunised children median sensitivities were 94, 95, and 83% and specificities 91, 95, and 97%. At the 10 mm threshold age correlated negatively with sensitivity of TST (*r* = −0.65, *P* = 0.04) and latitude correlated positively (*r* = 0.71, *P* = 0.02). *Conclusions*. For the 10 mm threshold the sensitivity of the TST is lower in BCG immunised children. Younger age and higher geographical latitude were associated with higher sensitivity of the TST.

## 1. Introduction

International guidelines differ in the threshold of tuberculin skin test (TST) induration regarded as indicating *Mycobacterium (M.) tuberculosis *infection in children with previous Bacillus Calmette-Guérin (BCG) immunisation. In the UK this is 15 mm and in the United States and Spain 10 mm [1]. The high risk posed by *M. tuberculosis* for children less than two years of age, with a risk of progression to disseminated tuberculosis and meningitis from 10 to 20% and tuberculosis in general of 50%, makes it essential to diagnose latent *M. tuberculosis* infection with maximum sensitivity. There is no gold standard test available to assess the sensitivity and specificity of tuberculin skin tests (TST) against in latent *M. tuberculosis *infection. Interferon gamma release assays (IGRA) have been shown to be more specific than TST particularly in BCG immunised persons [1] and may be more sensitive in children less than 2 years old and in low income countries where malnutrition and helminth and HIV infection are common [1–3]. Therefore the better performance characteristics of the IGRA compared to the TST make IGRA a suitable reference standard to determine the threshold of TST induration diameter used to define *M. tuberculosis *infection in children with and without BCG immunisation. This investigation adopted the methodology of a systematic review to address the following objectives:

1. to define the TST induration diameter with the best sensitivity and specificity in diagnosing *M. tuberculosis* infection compared to IGRA in children with and without previous BCG immunisation;
2. to determine the false negative rate of the TST with IGRA as reference standard for different TST induration thresholds;
3. to assess the age dependency of sensitivity and specificity of TST;
4. to assess the dependency of sensitivity and specificity of TST on geographical latitude.

## 2. Methods

### 2.1. Inclusion and Exclusion Criteria

Included were studies containing data on TST, IGRA, and BCG status of each participant in children (<16 years of age). Excluded were studies not allowing extraction of data on TST, IGRA, and BCG status in children or where results were only available for mixed adults and children groups. Excluded were data on patients with indeterminate IGRA.

### 2.2. Search Strategy

Data bases searched by the two authors independently were MEDLINE and Premedline (PubMed), EMBASE, and the Cochrane library using the key words: ELISPOT, QuantiFERON, interferon gamma release assay, IGRA, child, infant, newborn, neonate, and tuberculosis with a limit to: Human and English Language and Publication Year from 1993. Extracted were data on results for IGRA and TST and BCG immunisation, type of IGRA, procedure of TST, location of study, and proportion of children with active tuberculosis, comorbidities, and age. Documentation of the search strategy followed the PRISMA guidelines.

### 2.3. Data Analysis

#### 2.3.1. Comparison of Sensitivities and Specificities for Different Tuberculin Skin Test Induration Thresholds

Sensitivities and specificities of TST induration thresholds measured against IGRA result as reference standard were calculated from individual patient data where it is possible using software MedCalc Statistical Software 11.5.0 (MedCalc Software bvba, Ostend, Belgium).

Medians for sensitivities and specificities across induration thresholds were compared by Kruskal-Wallis test to take into account multiple comparisons. The Mann-Whitney *U* test was used for comparison of specificities and sensitivities. Parameters in patients with and without BCG immunisation were compared by Fisher's exact test where original data were available. Because of the importance of not missing *M. tuberculosis* infection in children the proportions of negative TST with a positive IGRA were also calculated. For studies which contained data for different induration thresholds for the same population these were compared by Fisher's exact test.

#### 2.3.2. Investigation of Inconsistency between Studies

Appropriate results were pooled in a meta-analysis to visualise and calculate heterogeneity with a summary statistic derived using the DerSimonian and Laird Random effects model where significant heterogeneity was detected using Meta-DISc 1.4 software (J. Zamora, V. Abraira, A. Muriel, KS. Khan, Coomarasamy A. Meta-DiSc: a software for meta-analysis of test accuracy data. BMC Medical Research Methodology 2006, 6:31). The *I* ^2^ statistic was used to quantify inconsistency (heterogeneity) of studies with *I* ^2^ > 40% representing moderate, >60% substantial, and >80% considerable inconsistency. Inconsistency (heterogeneity) of studies as documented in a meta-analysis was explored by investigation of an association of age, percentage of patients with active tuberculosis, and geographical latitude with sensitivities and specificities. Geographical latitude was investigated because BCG immunisation is known to have a lower impact on the immune response to tuberculin in areas closer to the equator. SPSS version 20 (SPSS Inc., Illinois, USA) was used to obtain correlations of age, latitude, and rates of active tuberculosis and specificities or sensitivities. Pearson's correlation coefficient and Spearman's rho were used for correlation analysis. Excel (Microsoft Office Excel (Microsoft Corporation, Seattle, WA, USA)) was used to generate graphic representations of sensitivities and specificities of different TST induration thresholds and the correlation of sensitivities and specificities and age or latitude. A *P* value of <0.05 was taken to indicate a statistically significant difference.

## 3. Results

### 3.1. Comparison of Sensitivities and Specificities for Different Tuberculin Skin Test Induration Thresholds

**Figure 1:**
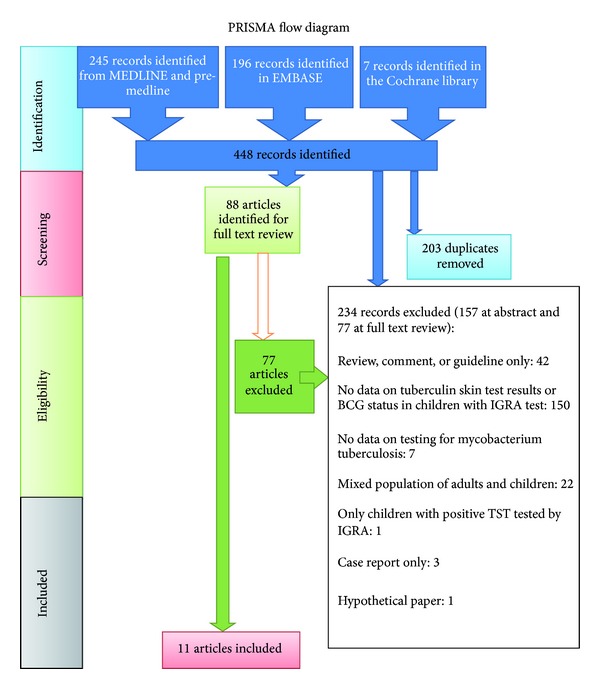
Study selection for systematic review—PRISMA flow diagram.

There were 245 records identified from PubMed, 196 in EMBASE, and 7 in the Cochrane library. 88 articles were retrieved for full text review and a total of 234 records were excluded (see Figure 1: PRISMA FLOW DIAGRAM). The 11 studies included had usable data on 2796 children (see Table 1). All studies investigated children as part of testing for *M. tuberculosis* infection. All but one study [4] used two tuberculin units of PPD RT 23 (Statens Serum Institute, Copenhagen, Denmark) for conduction of the tuberculin skin test. Eight studies used the QuantiFERON as IGRA, while three studies [1, 7, 13] used the ELISPOT version. Sensitivities, specificities, and percentages of false negative TST results for different TST induration thresholds were tabulated in Table 2.

In BCG immunised children 5, 10, and 15 mm induration thresholds had median (range) sensitivities of 87 (74.6 to 100.0), 70 (25 to 87), and 75 (60.9 to 83.6) %, respectively, and specificities of 67 (60.7 to 92.5), 93 (74.9 to 97.4), and 90 (70.7 to 97.0) %, respectively. In non-BCG immunised children median sensitivities were 94 (89 to 100), 95 (84 to 100), and 83.6% (one study only), respectively, and specificities were 91 (88.9 to 92.5), 95 (95 to 100), and 97% (one study only), respectively. There was no significant difference between sensitivities (*P* = 0.43) or specificities (*P* = 0.13) of induration thresholds with or without BCG immunisation (see Figures 4 and 5).

Comparison of sensitivities of induration thresholds 5, 10, and 15 mm with or without previous BCG immunisation is presented in Figure 4. There was no difference in sensitivities for a 5 or 15 mm induration threshold between patients with and without BCG immunisation (*P* = 0.26 and *P* = 0.50, resp.). For the 10 mm induration threshold sensitivities were significantly lower and hence false negative TST's were significantly more common (*P* = 0.014) in children with BCG immunisation.

For this 10 mm induration threshold very low sensitivities were reported in one study from Uganda [13] (25%) and one from South Africa [9] (33.3%). Analysis of the Ugandan study's anthropometric data showed a low level of under-nutrition, with prevalence of wasting being 5.4%. 13 (1.4%) of the participants were HIV infected, and the prevalence of other infections was as follows: asymptomatic malaria 4.0%, helminth infection 9.6%, and active tuberculosis 0.3%. In the South African study [9] all children had cancer: 61.8% haematological malignancies, 32.4% solid tumors, and 5.9% others.

One study with low specificity [1] (59.7%) was conducted in Istanbul, Turkey, with an ELISPOT, which was a precursor of the currently commercially available ELISPOT version. The other study showed a specificity of 60.8% [5] and was conducted in Seoul, South Korea, and 59.3% were clinically unwell with causes other than tuberculosis including pneumonia, chronic cough, fever of unknown origin, and lymphadenopathy.

### 3.2. Investigation of Inconsistency between Studies

**Figure 2:**
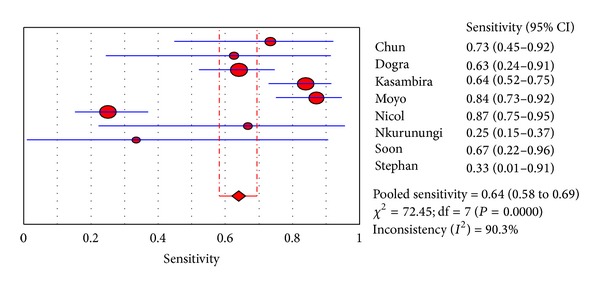
Meta-analysis of sensitivity of a 10 mm induration threshold of a tuberculin skin test in BCG immunised children (DerSimonian and Laird random effects model).

**Figure 3:**
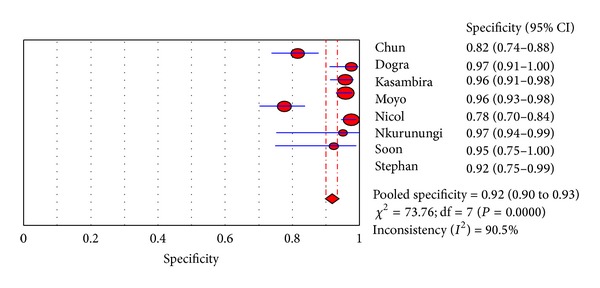
Meta-analysis of specificity of a 10 mm induration threshold of a tuberculin skin test in BCG immunized children (DerSimonian and Laird random effects model).

**Figure 4:**
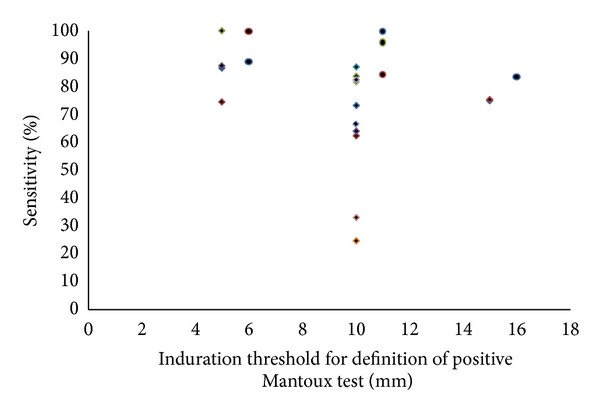
Sensitivity of different tuberculin skin test thresholds for detection of *M. tuberculosis* infection with (diamonds) and without (bullets) previous BCG immunisation with IGRA as a reference standard (each dot represents the result of one study).

**Figure 5:**
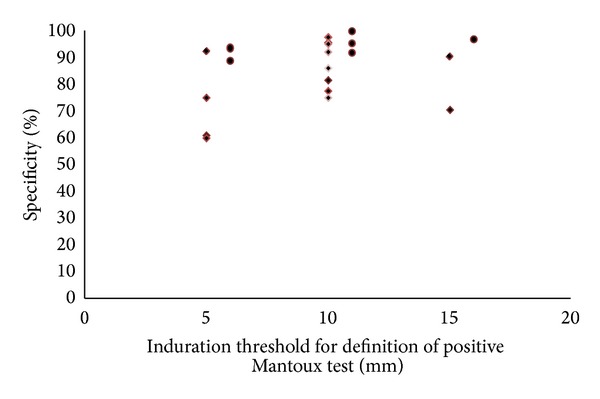
Specificity of different tuberculin skin test thresholds for detection of *M. tuberculosis *infection with (diamonds) and without (bullets) previous BCG immunisation with IGRA as a reference standard (each dots represents the result of one study).

For a 10 mm induration threshold in BCG immunised children a meta-analysis was employed to investigate for inconsistency (heterogeneity) between study results (see Figures 2 and 3). The studies Bakir 2009 and Mendez-Echevarria 2011 could not be included in the meta-analyses because the reports only allowed extraction of the sensitivities and specificities and not the raw data from which they were calculated. For other induration thresholds there were not enough studies available to allow for a meaningful meta-analysis to explore inconsistency between the majority of studies. The meta-analysis revealed considerable inconsistency (heterogeneity) of studies with regards to analysis of both sensitivities and specificities (see Figures 2 and 3).

We therefore explored potential sources of such inconsistency like presence of active tuberculosis, age, and geographical latitude.

The percentage of* M. tuberculosis* infected patients with features of active pulmonary tuberculosis did not correlate with sensitivity or specificity of the 10 mm threshold (*P* > 0.10).

**Figure 6:**
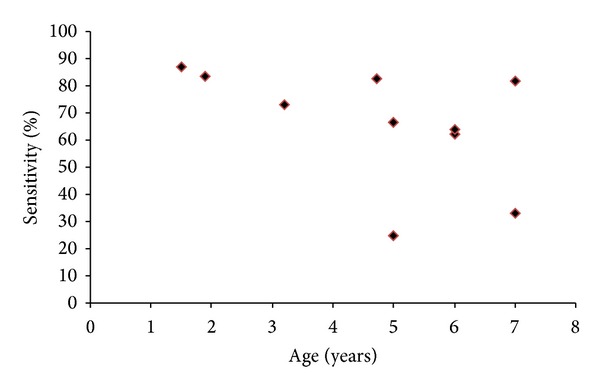
Sensitivity of a 10 mm threshold of induration of tuberculin skin test for detection of *M. tuberculosis* infection in relation to age in BCG immunised children using interferon gamma release assay as reference standard.

**Figure 7:**
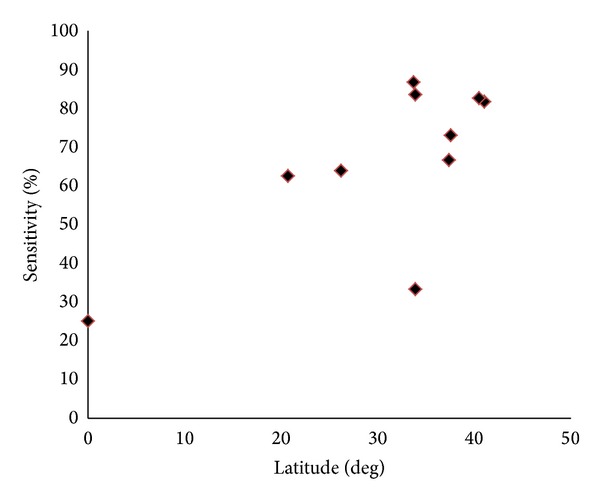
Correlation of sensitivity of a 10 mm tuberculin skin test induration threshold in BCG immunised children with geographical latitude of location where studies were conducted: each diamond shaped marker allocates a geographical latitude of the town where the study was located to the sensitivity of the 10 mm induration threshold for studies [1, 4, 5, 7–13].

Correlation analysis of sensitivity and age (Figure 6) or geographical latitude (Figure 7) was conducted for a subgroup of studies with data on an induration threshold of 10 mm and previous BCG immunisation. This subgroup was chosen as it was the only group with sufficient numbers to allow such an analysis.

Correlation of TST sensitivity with age (using an induration threshold of 10 mm) revealed a Spearman's rho correlation coefficient of −0.65 (*P* = 0.04) (Figure 6) indicating increased sensitivity of tuberculin skin testing relative to IGRA in younger children. Correlation of sensitivities with geographical latitude revealed a Pearson correlation coefficient of 0.71 (*P* = 0.02) indicating increased sensitivity of tuberculin skin testing with higher latitude (Figure 7).

Specificities for a threshold of 10 mm were equal or above 75% for all studies. There was no significant change in specificity at different ages or latitude.

### 3.3. Comparison of Sensitivity and Specificity of TST Induration Thresholds in BCG versus Not BCG Immunised Children

Three studies contained original data or results of analyses on children both with and without BCG immunisation.

The first [4] allowed direct statistical comparison of sensitivities and specificities in BCG immunised versus not immunised children. For a 10 mm induration threshold sensitivity in BCG immunised children was 62.5% (95% CI 24.49 to 91.48) and in nonimmunised children 100.0% (95% CI 29.24 to 100.0) (*P* = 0.49). Specificity was also not significantly different between those immunised: 97.44% (95% CI 91.04 to 99.69) and those not immunised: 100% (95% CI 79.41 to 100.0) (*P* = 0.90).

The second study by Bakir et al. [1] demonstrated at a TST induration threshold of 10 mm a sensitivity of 81.8% (95% CI 76.5 to 86.4) in BCG immunised people similar to 84.5% (95% CI 76.4 to 90.7) in nonimmunised children. Specificity was significantly lower: 74.9% (95% CI 70.4 to 79.1) in immunised compared to nonimmunised children 91.9% (95% CI 84.7 to 96.4).

In the third study by Méndez-Echevarría et al. [10] sensitivity was 82.6% (95% CI 63 to 93) in BCG immunised people compared to 95.9% (95% CI 88.6 to 98.6) in nonimmunised people and specificity was 86% (95% CI 80 to 90) in immunised people was significantly lower than in nonimmunised people with 95.5% (95% CI 91.1 to 97.8).

### 3.4. Comparison of False Negative TST Results with Different TST Induration Thresholds

In BCG immunised children 5, 10 and 15 mm induration thresholds had median (range) false negative rates of 13 (0.0 to 25.4), 30 (13 to 76), and 25 (16.4 to 39.1)%, respectively. In non-BCG immunised children median false negative rates were 6 (0.0 to 11), 5 (0.0 to 16), and 16.4%, respectively. There was no significant difference between false negative rates (*P* = 0.43) comparing different induration thresholds. The result was the same with or without BCG immunisation. For the 10 mm induration threshold the false negative rate was significantly higher in BCG immunised versus non-BCG immunised children (*P* = 0.014).

Two studies [5, 11] allowed comparisons of sensitivities and the corresponding false negative TST rates (Table 2) within the same study. For Chun et al. [5] these were not significantly different between TST induration thresholds of 5 and 10 mm (*P* = 0.65). For the study by Kasambira et al. [11] there was also no significant difference in sensitivity and the corresponding false negative TST rates between these induration thresholds (*P* = 0.21).

## 4. Discussion

### 4.1. Comparing Parameters Determining Diagnostic Accuracy between Induration Thresholds

This investigation showed that there were no significant differences in sensitivities or specificities between TST induration thresholds of 5, 10, or 15 mm or children with and without BCG immunization, but the number of studies was too small and they were too heterogenous to exclude a clinically significant difference. For the 10 mm threshold BCG immunised children had a lower sensitivity of the TST compared to non-immunized children, which may be due to comorbidities in the South African study [9] (oncology patients) and coinfections in the Ugandan study [13] affecting the TST more than the IGRA. Future studies should investigate whether TST induration threshold should be lower in BCG immunised children to optimise sensitivity.

### 4.2. The Significance of a False Negative Tuberculin Skin Test

The better correlation of IGRA versus TST with exposure in previous contact investigations particularly in BCG immunised populations was reflection of a higher specificity of IGRA compared to TST. It is thus not the rate of negative IGRA results amongst positive TST results which is clinically relevant but the rate of negative TST results amongst patients with positive IGRA. This latter rate is not likely to represent false positive IGRA results but indicates lower sensitivity of the TST. In view of the increased risk of progression to potentially fatal tuberculous meningitis in children less than two years old the 13 and 16% false negative TST rates at a 10 mm induration in BCG immunised populations with a median age of less than two years of this review [7, 12] suggest that both TST and IGRA should be used simultaneously to investigate *M. tuberculosis* infection if this threshold is used to define TST positivity. A subgroup analysis contained in another of the included studies [1] which did not break down the results according to BCG vaccination status was derived from 32 less than 2-year-old children and found false negative rates for a 5 mm TST induration threshold of 21.9%, for a 10 mm threshold 34.4%, and for a 15 mm threshold 37.5%. Application of an induration threshold of 15 mm for definition of a positive TST in people with previous BCG immunisation was associated with an unacceptably high rate of false negative TST from 24 to 39% in the studies reviewed.

### 4.3. Investigation of Causes of Inconsistency

Higher geographical latitude was associated with higher sensitivity of the tuberculin skin test in BCG immunised children. This may be due to the fact that BCG immunisation is associated with a reduced effect on the Th1 response to tuberculin at lower latitudes [14, 15]. This may explain the low sensitivity in the Ugandan study. Causes for this observation may include a Th2 dominated immune response associated with helminth infections or concomitant malnutrition reducing a Th1 dominated delayed hypersensitivity reaction. Increased sensitivities of the 10 mm TST threshold with younger age with the highest between 80 and 90% in children between one and two years of age may indicate that there is no reduction of TST sensitivity in younger children.

### 4.4. Implications for Clinical Practice and Future Research

The use of 5 and 15 mm induration thresholds in TSTs in guidelines for testing of young children for *M. tuberculosis* infection is currently not evidence based. The literature search did not find studies exclusively conducted on infants. The studies available for this review did not contain data on a 5 mm or 15 mm induration threshold for less than two-year-old children but because of the high risk associated with missing *M. tuberculosis* infection an induration threshold of 15 mm should not be used in children <2 years of age. IGRA testing should not be reserved for confirmatory testing in children less than two years of age for any TST induration threshold.

Future studies need to assess sensitivities of TST thresholds for infants measured against IGRA results and need to investigate systematically the influence of systemic inflammatory response syndromes associated with nontuberculous infections and haematological malignancies on the sensitivity of TST compared to IGRA. The influence of BCG immunisation on TST specificity needs to be investigated for all induration thresholds to determine which threshold optimises specificity without compromising sensitivity for all age groups.

## 5. Conclusions

For the 10 mm threshold there was a lower sensitivity of the TST in BCG immunised children.

Younger age and higher latitude were associated with higher sensitivity of the tuberculin skin test.

## Figures and Tables

**Table 1:** Characteristics of studies reporting on children with results on tuberculin skin test and interferon gamma release assay and information on BCG immunisation status (TB: tuberculosis).

Study reference	Country	Setting	Reason for testing	Age	Children with BCG immunisation	Children without BCG immunisation	Active TB (%)
Dogra et al. 2007 [4]^a^	India	Rural Medical School	Suspect cases or contacts	Median: 6.0 years (range 1 to 12)	86	19	10.5
Chun et al. 2008 [5]	South Korea	Municipal Children's Hospital	Close and casual contacts and cases	Median 3.2 years (range 0–15.8)	145	—	3.0
Taylor et al. 2008 [6]	United Kingdom	Municipal Hospital	Contacts	Mean 10 years (range 4 to 16)	45	—	10.9 (suspected)
Nicol et al. 2009 [7]	South Africa	Semirural population	Suspected tuberculosis or contacts	Median 1.5 years (IQR 1.1 to 2)	214	—	26.8
Bakir et al. 2009 [1]	Turkey	Municipal clinics	Contacts	Median 7 years (IQR 3 to 11)	652	209	1.3
Soon et al. 2010 [8]	South Korea	Municipal Hospital	Contacts	Median 5 years	26	—	21.2
Stefan et al. 2010 [9]	South Africa	Municipal Hospital	Children newly diagnosed with cancer	Median 7 years (range 0.1 to 15)	29	—	2.9
Méndez- Echevarría et al. 2011 [10]	Spain	Municipal Hospitals	Immigrants from endemic areas, contacts, and cases	Mean age 4.73 (range 0.1 to 15)	213	246	14.8
Kasambira et al. 2011 [11]	South Africa	Rural clinics, District Hospital	Contacts	Median age 6 years (IQR 3 to 9)	236	—	19.0
Moyo et al. 2011 [12]	South Africa	Rural district	Contacts and suspected cases	Median 1.9 years (range 0.75 to 2.83)	376	—	13.1
Nkurunungi et al. 2012 [13]	Uganda	Municipal Hospital	Population based screening	5 years	300	—	0.3

^a^Numbers in brackets refer to the number of the reference in References of the paper.

**Table 2:** Sensitivities and specificities of tuberculin skin test thresholds and BCG immunisation status.

Study reference	BCG immunisation status (immunised +, not immunised −)	Sensitivity (95% confidence interval)	Specificity (95% confidence interval)	False negative rate as percentage (95% confidence interval) of people with negative tuberculin skin test amongst IGRA positive patients
TST induration threshold 5 mm				
Chun et al. 2008 [5]^a^	+	86.7 (59.5 to 98.3)	60.8 (51.8 to 69.2)	13.3 (1.7 to 40.5)
Bakir et al. 2009 [1]	+	87.4 (82.6 to 91.2)	59.7 (54.7 to 64.5)	12.6 (8.8 to 17.4)
	−	89.1 (81.7 to 94.2)	88.9 (81.0 to 94.3)	10.9 (5.8 to 18.3)
Soon et al. 2010 [8]	+	100.0 (54.1 to 100.0)	75.00 (50.9 to 91.3)	0.0 (0.0 to 45.9)
Méndez-Echevarría et al. 2011 [10]	−	100.0 (95.0 to 100.0)	93.6 ( 88.7 to 96.5)	0.0 (0.0 to 5.0)
Kasambira et al. 2011 [11]	+	74.7 (63.3 to 84.0)	92.5 (87.3 to 96.1)	25.3 (16.0 to 36.7)

TST induration threshold 10 mm				
Dogra et al. 2007 [4]	+	62.5 (24.5 to 91.5)	97.4 (91.0 to 99.7)	37.5 (8.5 to 75.5)
	−	100.0 (29.2 to 100.0)	100.0 (79.4 to 100.0)	0.0 (0.0 to 70.8)
Chun et al. 2008 [5]	+	73.3 (44.9 to 92.2)	81.5 (73.8 to 87.8)	26.6 (7.8 to 55.1)
Bakir et al. 2009 [1]	+	81.8 (76.5 to 86.4)	74.9 (70.4 to 79.1)	18.2 (13.6 to 23.5)
	−	84.5 (76.4 to 90.7)	91.9 (84.7 to 96.4)	15.5 (9.3 to 23.6)
Nicol et al. 2009 [7]	+	87.0 (75.1 to 94.6)	77.5 (70.2 to 83.7)	13.0 (5.4 to 24.9)
Soon et al. 2010 [8]	+	66.7 (22.3 to 95.7)	95.0 (75.1 to 99.8)	33.3 (4.3 to 77.7)
Stefan et al. 2010 [9]	+	33.3 (0.008 to 90.6)	92.3 (74.9 to 99.0)	66.6 (9.4 to 99.2)
Kasambira et al. 2011 [11]	+	64.0 (52.1 to 74.8)	95.6 (91.2 to 98.2)	36.0 (25.2 to 47.9)
Méndez-Echevarría et al. 2011 [10]	+	82.6 (63.0 to 93.0)	86.0 (80.0 to 90.0)	17.4 (7.0 to 37.0)
	−	95.9 (88.6 to 98.6)	95.5 (91.1 to 97.8)	4.1 (1.4 to 11.4)
Moyo et al. 2011 [12]	+	83.8 (72.9 to 91.6)	95.8 (92.9 to 97.7)	16.1 (8.4 to 27.1)
Nkurunungi et al. 2012 [13]	+	25.0 (15.3 to 36.9)	97.4 (94.5 to 99.0)	75.0 (63.1 to 84.7)

TST induration threshold 15 mm				
Taylor et al. 2008 [6]	+	75.0 (19.4 to 99.4)	70.7 (54.5 to 83.9)	25.0 (0.6 to 80.6)
Bakir et al. 2009 [1]	+	75.5 (69.7 to 80.7)	90.5 (87.2 to 93.2)	24.5 (19.3 to 30.3)
	−	83.6 (75.4 to 90.0)	97.0 (91.4 to 99.4)	16.4 (10.0 to 24.6)
Méndez-Echevarría et al. 2011 [10]	+	60.9 (40.8 to 77.8)	97.0 (92.4 to 98.2)	39.1 (22.2 to 59.2)

^a^Numbers refer to the number of the reference in References of the paper.
